# Low CCL19 expression is associated with adverse clinical outcomes for follicular lymphoma patients treated with chemoimmunotherapy

**DOI:** 10.1186/s12967-021-03078-9

**Published:** 2021-09-20

**Authors:** Yu Zhou, Shasha Wang, Yunxia Tao, Haizhu Chen, Yan Qin, Xiaohui He, Shengyu Zhou, Peng Liu, Jianliang Yang, Sheng Yang, Lin Gui, Ning Lou, Zhishang Zhang, Jiarui Yao, Xiaohong Han, Yuankai Shi

**Affiliations:** 1 Department of Medical Oncology, National Cancer Center/National Clinical Research Center for Cancer/Cancer Hospital, Chinese Academy of Medical Sciences & Peking Union Medical College, Beijing Key Laboratory of Clinical Study on Anticancer Molecular Targeted Drugs, No. 17 Panjiayuan Nanli, Chaoyang District, Beijing, 100021 China; 2 Clinical Pharmacology Research Center, Peking Union Medical College Hospital, State Key Laboratory of Complex Severe and Rare Diseases, NMPA Key Laboratory for Clinical Research and Evaluation of Drug, Beijing Key Laboratory of Clinical PK & PD Investigation for Innovative Drugs, Chinese Academy of Medical Sciences & Peking Union Medical College, No. 41 Damucang Hutong, Xicheng District, Beijing, 100032 China

**Keywords:** Follicular lymphoma, CCL19, Prognosis, Survival outcome, Chemokine

## Abstract

**Background:**

This study aimed to recognize the hub genes associated with prognosis in follicular lymphoma (FL) treated with first-line rituximab combined with chemotherapy.

**Method:**

RNA sequencing data of dataset GSE65135 (n = 24) were included in differentially expressed genes (DEGs) analysis. Weighted gene co-expression network analysis (WGCNA) was applied for exploring the coexpression network and identifying hub genes. Validation of hub genes expression and prognosis were applied in dataset GSE119214 (n = 137) and independent patient cohort from Cancer Hospital, Chinese Academy of Medical Sciences & Peking Union Medical College (n = 32), respectively, by analyzing RNAseq expression data and serum protein concentration quantified by ELISA. The Gene Set Enrichment Analysis (GSEA), gene ontology (GO) and Kyoto Encyclopedia of Genes and Genomes (KEGG) pathway enrichments analysis were performed. CIBERSORT was applied for tumor-infiltrating immune cells (TIICs) subset analysis.

**Results:**

A total of 3260 DEGs were obtained, with 1861 genes upregulated and 1399 genes downregulated. Using WGCNA, eight hub genes, *PLA2G2D*, *MMP9*, *PTGDS*, *CCL19*, *NFIB*, *YAP1*, *RGL1*, and *TIMP3* were identified. Kaplan–Meier analysis and multivariate COX regression analysis indicated that *CCL19* independently associated with overall survival (OS) for FL patients treated with rituximab and chemotherapy (HR = 0.47, 95% CI [0.25–0.86], p = 0.014). Higher serum CCL19 concentration was associated with longer progression-free survival (PFS, p = 0.014) and OS (p = 0.039). TIICs subset analysis showed that CCL19 expression had a positive correlation with monocytes and macrophages M1, and a negative correlation with naïve B cells and plasma cells.

**Conclusion:**

*CCL19* expression was associated with survival outcomes and might be a potential prognostic biomarker for FL treated with first-line chemoimmunotherapy.

**Supplementary Information:**

The online version contains supplementary material available at 10.1186/s12967-021-03078-9.

## Introduction

Follicular lymphoma (FL) is the second common B-cell lymphoma that compromises about 12% of all mature non-Hodgkin lymphoma (NHL) [1, 2]. FL is an indolent NHL and is characterized with repeatedly relapse in the long disease history in most patients [3]. Although the advent of rituximab improved the progression-free survival (PFS) and overall survival (OS) of FL [4, 5], still approximately 20% patients with FL relapsed within two years after first-line chemoimmunotherapy [6], which lead to a dismal prognosis. Therefore, identifying the high-risk patients with suboptimal treatment response before treatment and improving their survival outcomes are necessary for the management of FL.

Most previous studies focused on screening differential expression genes and establishing gene models in FL [7, 8]. However, the interconnectivity and co-expression of the genes was ignored. Weighted gene co-expression network analysis (WGCNA) is a useful method to explore the interactions and relationships within genes and can help to recognize the hub genes associated with clinical characteristics [9, 10]. WGCNA can recognize the core gene and provide information for potential clinical prognosis biomarkers and has been widely used in cancer genome-related researches, including other subtypes of lymphoma [11–13]. In this study, we sought to identify the hub genes for FL using WGCNA conducted in a cohort containing FL samples and normal samples. We focused on the prognosis value of the identified hub genes. The association of hub genes and survival outcomes was validated in two independent cohorts including both tumor samples and serum samples of FL patients receiving standard first-line treatment, respectively. We aimed to raise new potential biomarkers for FL, which could bring more insight for diagnosis and clinical treatment in FL.

## Materials and methods

### Patients and datasets

GSE65135 containing RNA sequencing (RNA-seq) data of 24 cases, including 10 normal tonsil tissue (control group) and 14 FL tissue (cancer group) was obtained from Gene Expression Omnibus (GEO) database (https://www.ncbi.nlm.nih.gov/geo/) and used for exploring the differential expressed genes (DEGs). GPL570 (Affymetrix Human Genome U133 Plus 2.0 Array) was used for this dataset and the annotation for the gene IDs was conducted using the hgu133plus2.db package in R software (Vienna, Austria. https://www.R-project.org/). The Affy package in R was applied for raw data quality control, preconditioning and sorting out [14]. The expression of repeated genes was expressed as mean values.

To validate the prognostic value of hub genes identified from GSE65135, GEO database was searched using the term “follicular lymphoma”, with organism restricted to “homo sapiens”. Up to January 9th, 2021, a total of 147 FL related GEO series was screened. Datasets of expression data with complete prognostic information were included in the validation cohort. Only one dataset, GSE119214, contained expression data and survival data (failure-free survival and OS) of pre-treated formalin-fixed and paraffin-embedded samples of 137 FL patients treated with rituximab and chemotherapy and was included in the validation cohort. GPL13938 (Illumina HumanHT-12 WG-DASL V4.0 expression beadchip) was selected for this microarray dataset and gene IDs were mapped to the microarray probes using the annotation information provided by GEO dataset.

Additionally, pre-treated serum samples of patients with FL treated with first-line chemoimmunotherapy in the Cancer Hospital, Chinese Academy of Medical Sciences & Peking Union Medical College (CAMS & PUMC) from 2014 to 2018 were used for further validation of hub genes with clinical significance identified from GSE119214. Inclusion criteria including: patients with newly diagnosed FL, receiving standard first-line R-CHOP (rituximab plus cyclophosphamide, doxorubicin, vincristine, and prednisone) or R-CHOP-like chemoimmunotherapy regimen, with follow-up information. All the samples were obtained before the initiation of treatment and were with informed consent. Sample collection was approved by the Hospital’s Protection of Human Subjects Committee. All data collected were anonymized. This study was approved by the medical ethics committee of Cancer Hospital, CAMS & PUMC (No. 19/088-1873). The cutoff date for follow-up was July 2nd, 2021. A total of 32 samples were included in this cohort (CHCAMS cohort). The patient characteristics of GSE119214 and CHCAMS cohort were shown in Additional file 1: Table S1.

### Differentially expressed genes screening

The limma package in R was used for screening the DEGs between control group and cancer group in GSE65135 [15]. The thresholds for DEGs were set as follows: (1) p < 0.05; (2) log2 (fold change) > 1 or < − 1. Volcano plot and heatmap generated by the ggplot2 package were displayed to show the DEGs.

### Gene enrichment analysis

Gene Ontology (GO) analysis was performed for analyzing the unique biological significance based on DEGs. The Kyoto Encyclopedia of Genes and Genomes (KEGG) analysis was performed to find the important pathways among the DEGs. The clusterProfiler package in R was used for analyzing GO annotation and KEGG pathway [16]. Gene Set Enrichment Analysis (GSEA) was applied to analyze the signal pathway enriched in tumor samples [17]. The GOplot package in R was used for result visualization [18].

### Coexpression network construction and hub gene selection

WGCNA package in R was applied for exploring the coexpression network and identifying hub genes [9, 10]. Expression data of DEGs were input into R and undergoing quality check before coexpression analysis. Samples and genes with poor quality would be excluded. Expression data of qualified samples were included in the analysis and similar matrix was constructed by calculating the Pearson correlation coefficient of two genes. Soft thresholding power was explored to ensure a scale-free network. The mean connectivity and scale independence of network modules were analyzed using gradient under soft thresholding power ranging from 1 to 20. Hierarchical clustering dendrogram summarized the gene modules. The minimum number of each gene module was set at 50 to ensure the reliability of each module. Heatmap and topological overlap matrix (TOM) plot were drawn to display the intensity of interaction among the modules.

The genes of modules were then used to construct the functional protein network. To further analyze the hub genes of the network, edges and nodes of network were output under certain threshold value (0.4) to Cytoscape software (version 3.8.0; http://www.cytoscape.org/) for analyzing and visualizing [19]. CytoHubba plugin in Cytoscape was applied for analyzing the degree of connectivity for each gene. The degree of connectivity set to rank genes. Genes with connectivity degree over 20 were identified and were considered to be hub genes. Identified hub genes were uploaded in the Search Tool for the Retrieval of Interacting Genes/Proteins (STRING) online database (version 11.0, https://string-db.org/) for analyzing the protein–protein interaction (PPI). Involved pathways of identified hub genes were explored in KEGG website (https://www.kegg.jp/kegg/).

### Validation of hub genes

To validate the prognostic power of hub genes, Kaplan–Meier analysis were applied using the data of GSE119214 and CHCAMS cohort. The best expression cut-off value for each hub gene for OS was obtained by the maxstat package in R. To identify the independent prognostic value of hub genes, multivariate analysis was applied.

To further explore mRNA levels of hub genes, ONCOMINE database (http://www.oncomine.org), a publicly accessible online cancer microarray database that facilitates the discovery of genome wide expression analyses, was applied. Student’s t test was conducted for comparing the mRNA level of cancer specimens and normal control datasets. Fold change value was set as 2 and p value < 0.05 were considered as significant.

### Measurement of serum CCL19 concentration

Peripheral blood samples were obtained before first-line treatment, and serum was extracted and stored at − 80 °C until use. Serum CCL19 concentrations were quantified using the CCL19 human enzyme-linked immunosorbent assay (ELISA) kit according to the manufacturer’s instructions (Mlbio, Enzyme-linked Biotechnology Co., Shanghai, China).

### CIBERSORT analysis

CIBERSORT (https://cibersort.stanford.edu/) was applied to analyze the association of tumor microenvironment and the expression of CCL19 [20]. The proportion of tumor-infiltrating immune cells (TIICs) of each samples in GSE119214, including naive B cells, memory B cells, plasma cells, CD8+ T cells, naive CD4+ T cells, CD4+ resting memory T cells, CD4+ memory-activated T cells, follicular helper T cells, Treg cells, γδ T cells, resting natural killer cells, activated natural killer cells, monocytes, M0 macrophages, M1 macrophages, M2 macrophages, resting dendritic cells, activated dendritic cells, resting mast cells, activated mast cells, eosinophils, and neutrophils, was calculated.

### Statistical analysis

Univariable analysis was performed using Kaplan–Meier survival analysis with log-rank test. Multivariable analysis was performed using COX regression model. Correlations between two groups were calculated with Spearman’s coefficient (*R*). Comparison of two groups was performed using Mann–Whitney–Wilcoxon test and comparison of multiple groups was performed using Kruskal–Wallis test. All statistical analyses were performed and visualized by R studio software (version 4.0.3, https://www.r-project.org/), GSEA software (version 4.1.0, http://www.gsea-msigdb.org/gsea/) and GraphPad PRISM (version 8.0.2). Two-side p value < 0.05 was considered as statistically significant.

## Result

### Overview of differentially expressed genes

**Fig. 1:**
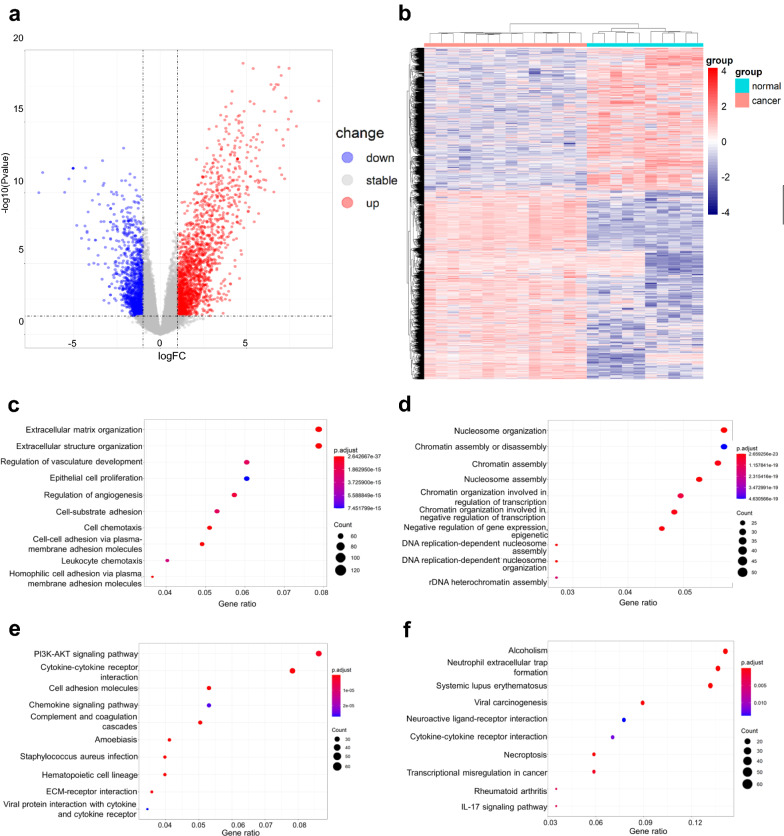
Differential expression profile and enrichment analysis result of GSE65135. **A** Volcano plot of all differentially expressed genes (DEGs); **B** Heatmap of all DEGs between 14 tumor samples (red) and 10 normal samples (blue); **C** Top 10 Gene Ontology (GO) terms of upregulated DEGs; **D** Top 10 GO terms of downregulated DEGs; **E** Top 10 Kyoto Encyclopedia of Genes and Genomes (KEGG) pathways of upregulated DEGs; **F** Top 10 KEGG pathways of downregulated DEGs

In differential expression analysis, a total of 3260 DEGs were obtained, of which 1861 genes were upregulated and 1399 genes were downregulated in tumor samples compared with normal samples (Fig. 1a, b).

To further analyze the biological function of the DEGs, the upregulated and downregulated genes were further analyzed with GO and KEGG enrichment analysis, respectively. Functional enrichment analysis was performed in the biological process. Up-regulated DEGs were enriched in extracellular matrix organization, extracellular structure organization and regulation of vasculature development, while down-regulated DEGs were enriched in nucleosome assembly, chromatin assembly or disassembly and chromatin assembly (Fig. 1c and d). KEGG analysis showed that the upregulated DEGs were enriched in PI3K-AKT signaling pathway, cytokine-cytokine receptor interaction and cell adhesion molecules. The down-regulated DEGs were involved in alcoholism, neutrophil extracellular trap formation and systemic lupus erythematosus (Fig. 1e and f).

As all the genes included in the GO and KEGG analysis were selected under the threshold set artificially (p < 0.05; log2 [fold change] > 1 or < − 1), the results might be different under different thresholds. Therefore, all expression dataset was included in GSEA analysis. Results showed that IL-6/JAK/STAT3 signaling, complement and mitotic spindle were three pathways that mostly enriched in tumor samples (Additional file 1: Fig. S1).

### Weighted gene correlation network analysis

**Fig. 2:**
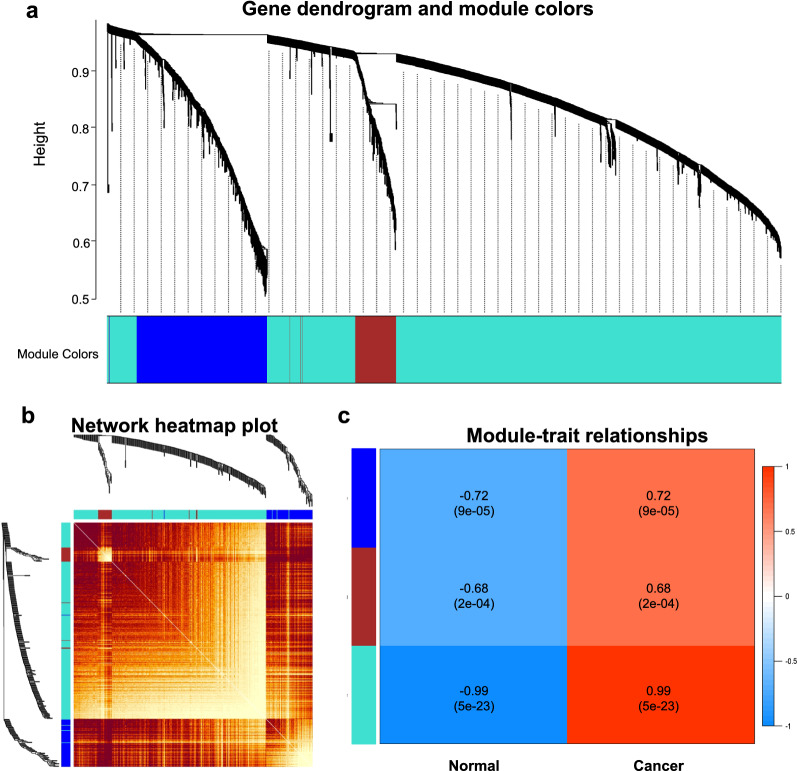
Weighted gene co-expression network analysis (WGCNA) for all DEGs identified from GSE65135. **A** Hierarchical cluster tree of co-expression modules identified by WGCNA. **B** Heatmap of topological overlap matrix (TOM); **C** Relationship of modules and cancer

To explore the key modules and hub genes in FL, WGCNA was performed for network construction to find highly-correlated genes. All the cancer samples and controlled samples were included in the analysis after quality check (Additional file 1: Fig. S2). Soft thresholding power was set at 6 to ensure a scale-free network, with scale-free *R*^*2*^ = 0.88 and mean *k* = 134 (Additional file 1: Fig. S3). Gene modules were explored and the identified modules were showed in the hierarchical cluster tree (Fig. 2a). A total of 3260 DEGs were allocated in three modules, with 633 genes in blue gene module, 209 genes in brown gene module and 2418 genes in turquoise gene module. Heatmap plot showed the TOM among all genes and the interactive relationships between all three coexpression modules (Fig. 2b). The relationship of the modules with FL was illustrated in Fig. 2c. According to the Pierson correlation coefficient between a module and sample feature for each module, turquoise module was closely associated with FL (*R* = 0.99) and the genes of this module were further analyzed. The functional enrichment of genes in turquoise module was further explored. GO analysis showed that cell chemotaxis, extracellular matrix organization and extracellular structure organization were the most enriched pathways of genes in turquoise module. KEGG analysis indicated that genes of turquoise module were mostly enriched in alcoholism, neutrophil extracellular trap formation and systemic lupus erythematosus pathways (Additional file 1: Fig. S4).

### Identification of hub genes

The threshold of edge weight was set to be more than 0.4 to localized hub genes. A total of 1791 edges and 382 nodes of turquoise module were included in Cytoscape analysis. The genes involved in the network were ranked by degree calculated in cytoHubba plugin to explore potential hub genes. Additional file 1: Fig. S5a showed the top 50 genes ranked by degree in turquoise module. The top eight genes with a threshold degree > 20 were identified and considered as hub genes (*PLA2G2D*, *MMP9*, *PTGDS*, *CCL19*, *NFIB*, *YAP1*, *RGL1*, and *TIMP3*). The PPI network of hub genes was illustrated in Additional file 1: Fig. S5b.

### RNA expression analysis for the validation of hub genes

Oncomine database was applied to analyze the mRNA expression of the eight hub genes. Additional file 1: Fig. S6 illustrated the expression of the hub genes among different cancer types. All of the eight hub genes were overexpressed and *MMP9*, *NFIB*, *YAP1*, *RGL1* and *TIMP3* expression were down-regulated across different datasets.

**Fig. 3:**
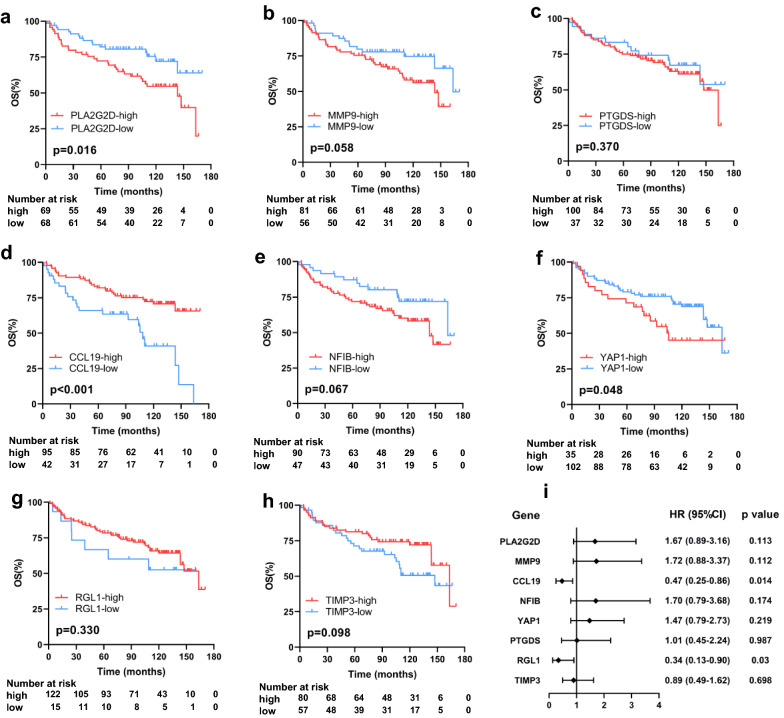
Kaplan–Meier survival analysis and multivariate analysis of hub genes in GSE119214. **A** PLA2G2D; **B** MMP9; **C** PTGDS; **D** CCL19; **E** NFIB; **F** YAP1; **G** RGL1; **H** TIMP3; **I** forest plot of multivariate Cox analysis result

Next, the expression and prognostic value of hub genes were validated in another independent GEO dataset with expression profiling and survival information of 137 FL samples (GSE119214) [21]. The whole 137 patients were all received rituximab in combination with chemotherapy as first-line treatment. The detailed baseline patient characteristics and survival information were shown in Additional file 1: Table S1. Kaplan–Meier analysis was performed and *PLA2G2D*, *CCL19*, and *YAP1* were found to be significantly associated with OS while the expression of other five genes (*MMP9*, *PTGDS*, *NFIB*, *RGL1*, and *TIMP3*) showed no significant relationship with OS (Fig. 3a–h and Additional file 1: Fig. S7). To determine independent prognostic genes, multivariate COX regression analysis was performed among the eight hub genes. Higher expression of *CCL19* and *RGL1* were significantly associated with longer OS (HR = 0.47, 95% CI [0.25–0.86], p = 0.014 for *CCL19*; HR = 0.34, 95% CI [0.13–0.90], p = 0.03 for *RGL1*) (Fig. 3i).

**Fig. 4:**
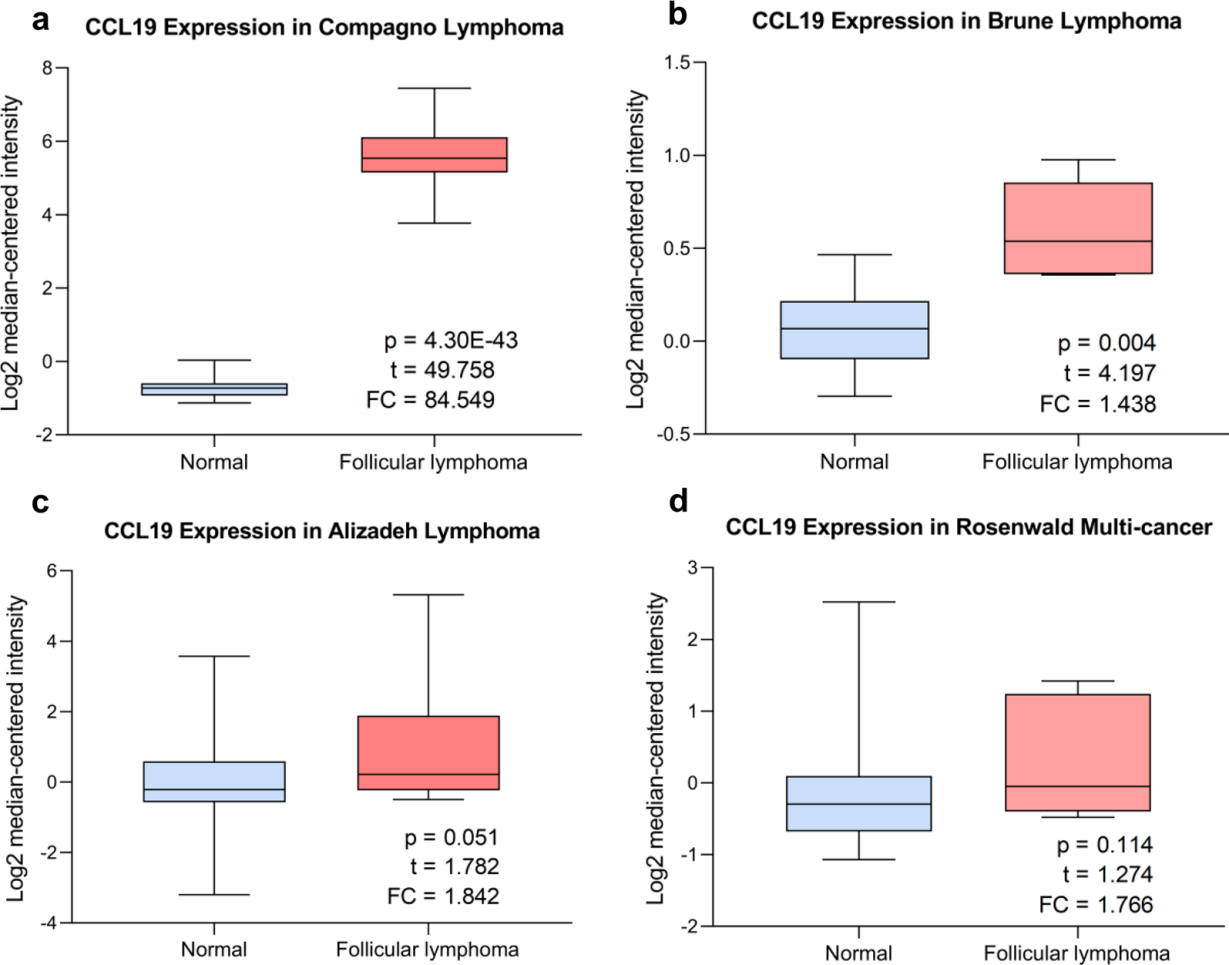
Box plots of CCL19 mRNA expression in Oncomine database comparing follicular lymphoma and normal tissue (**A**–**D**)

Overall, 137 samples in GSE119214 were grouped into *CCL19* high expression group and *CCL19* low expression group and were further included in GSEA to explore the pathways enriched in *CCL19* high expression and low expression groups. Results showed that in *CCL19* low expression group, genes were enriched in pathways of spliceosome, protein export and ubiquitin mediated proteolysis, while genes in *CCL19* high expression group were enriched in IL6/JAK/STAT3 signaling pathway, KRAS signaling up and complement (Additional file 1: Fig. S8). As *CCL19* showed independent prognostic value in both Kaplan–Meier analysis and multivariate COX analysis in the GEO dataset, the expression of *CCL19* was further explored. Oncomine dataset analysis indicated that mRNA of *CCL19* was ≥ 84.549 and ≥ 1.438 fold elevated in FL samples compared to normal tissue in Compagno lymphoma dataset and Brune lymphoma dataset, respectively (Fig. 4a and b). However, in Alizadeh lymphoma dataset and Rosenwald multi-cancer dataset, the expression level of *CCL19* was showed no statistical significance between FL samples and normal tissue (Fig. 4c and d).

### Serum CCL19 concentration measurement

**Fig. 5:**
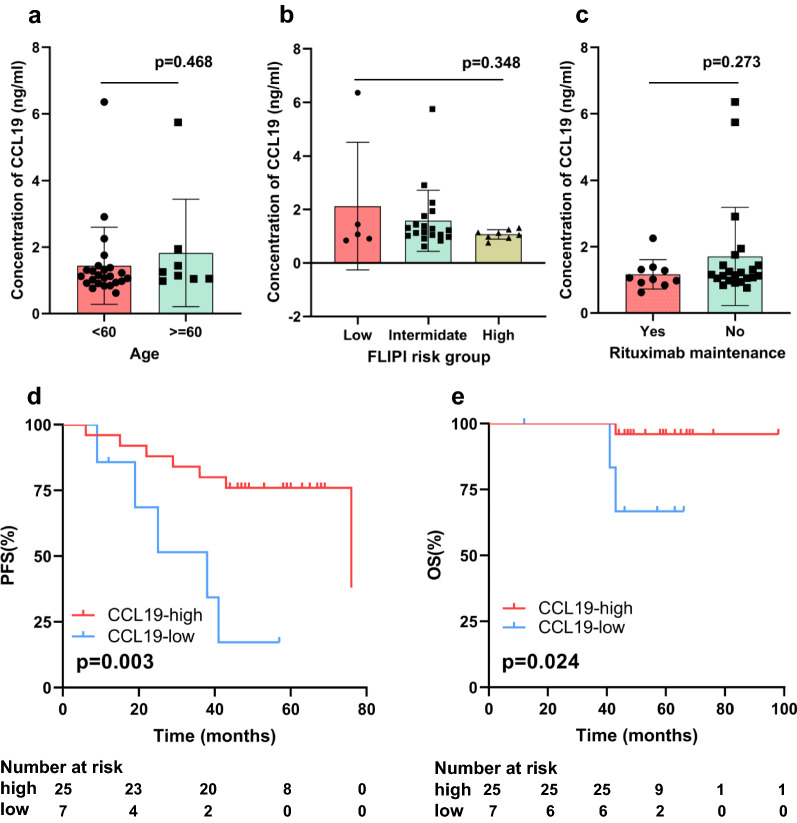
The correlation of CCL19 concentration with clinical characteristics and survival outcomes in CHCAMS cohort. Concentration of serum CCL19 among difference age groups (**A**), different FLIPI groups (**B**) and different rituximab maintenance groups (**C**). Kaplan–Meier curves of CCL19 concentration with **D** progression-free survival and **E** overall survival. FLIPI: Follicular Lymphoma International Prognostic Index; PFS: progression-free survival; OS: overall survival

The prognostic value of *CCL19* was further validated in CHCAMS cohort. A total of 32 pre-treatment serum samples of treatment naïve FL were included in the ELISA test, with 16 (50%) patients were male. The median age was 47 years old. All the patients received first-line chemoimmunotherapy, including R-CHOP or R-CHOP-like regimens, with 10 of 32 (31.2%) receiving rituximab maintenance. Median follow-up time was 58.0 (range: 12.0–98.0) months. The detailed baseline patient characteristics and survival information were shown in Additional file 1: Table S1. There were no difference of serum CCL19 concentration between difference age groups (mean concentration: 1.439 ng/ml in age over 60 years vs. 1.823 ng/ml in age less than 60 years, p = 0.468), Follicular Lymphoma International Prognostic Index risk groups (mean concentration: 2.122 ng/ml in low-risk group vs. 1.578 ng/ml in intermediated-group vs. 1.070 ng/ml in high-risk group, p = 0.348) and rituximab maintenance groups (mean concentration: 1.165 ng/ml in maintenance group vs. 1.578 ng/ml in no maintenance group, p = 0.278) (Fig. 5a–c). The best-cutoff value for serum CCL19 was 0.94 ng/ml (Additional file 1: Fig. S8) and patients were grouped into CCL19 high concentration and CCL19 low concentration groups according to the cutoff value. Kaplan–Meier curves showed that serum CCL19 concentration was associated with PFS and OS, with CCL19 high concentration group having longer PFS (p = 0.014) and OS (p = 0.039) (Fig. 5d and f) comparing with CCL19 low concentration group. CCL19 concentration, rituximab maintenance and FLIPI risk group were included in the multivariate COX analysis for PFS. Results indicated that high concentration of CCL19 was the independent factor for PFS (HR = 0.14, 95% CI [0.03–0.58], p = 0.007).

### CIBERSORT analysis

**Fig. 6:**
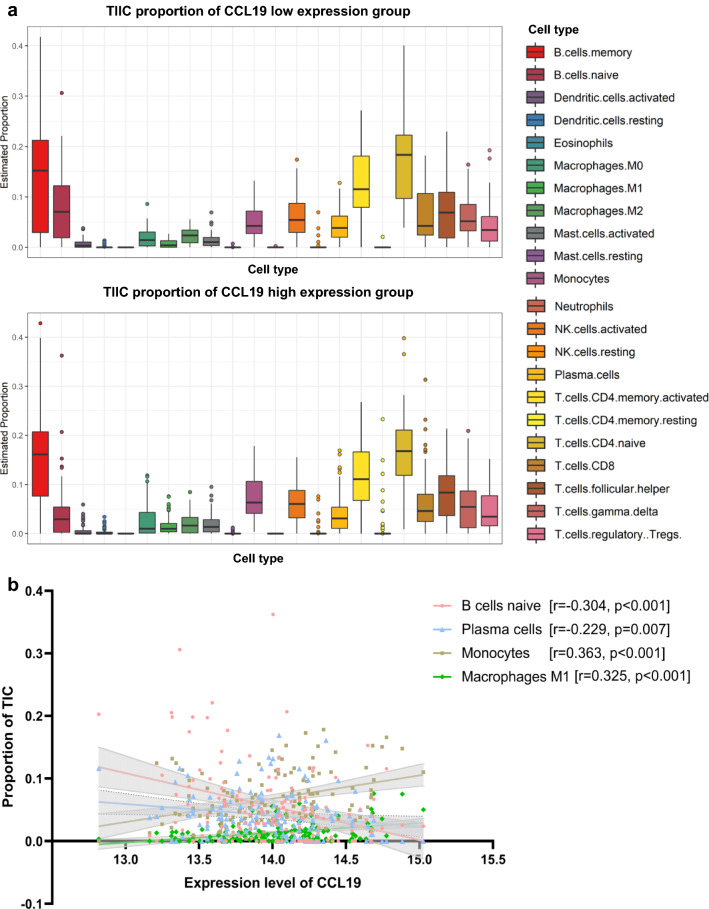
Results of tumor-infiltrating immune cell analysis using CIBERSORT. **A** The proportion of tumor-infiltrating immune cell (TIIC) in CCL19 low-expression (above) and high-expression (below) groups. **B** Correlation of TIIC subtypes and CCL19 expression. TIIC: tumor-infiltrating immune cell

To further elucidate which subset of TIICs might serve as regulatory role in different *CCL19* expression groups, CIBERSORT analysis was applied in dataset GSE119214 to calculate the relationship of *CCL19* expression and microenvironment cell subsets. The proportion of different subsets of TIICs between *CCL19* low expression and high expression groups was showed in Fig. 6a. The correlations were further quantified and *CCL19* mainly had a positive correlation with monocytes (*R* = 0.363, p < 0.001) and macrophages M1 (*R* = 0.325, p < 0.001), and it had a negative correlation with naïve B cells (*R* = − 0.304, p < 0.001) and plasma cells (*R* = − 0.229, p = 0.007, Fig. 6b).

## Discussion

FL is a kind of indolent lymphoma with heterogeneity. Previous studies focused on identifying related genes using differential expression analysis. In this study, we applied WGCNA analysis utilizing RNAseq data of FL and found three related co-expression gene modules, explored the gene network relationship, and identified eight key genes of the network. The prognostic value of eight key genes were validated in two independent FL patient cohorts and we identified that *CCL19* was significantly associated with PFS and OS in FL patients receiving first-line chemoimmunotherapy, which indicated that *CCL19* could be a potential prognosis biomarker for FL. To our best knowledge, this study is the first to report the potential prognostic value of *CCL19* in FL.

In previous studies, different genes were reported to be related to the development of FL. About over 85% FL patients were characterized by t (14;18), which resulted in the overexpression of BCL2 protein, a family of protein that inhibits cell apoptosis [22]. Mutations of chromatin modifying genes, including *EZH2*, *KMT2D* and *CREBBP*, were reported to play a complex role in FL [23]. Mutations in linker histone genes (HIST1H1 B, C, D, and E; OCT2 [POU2F2]; IRF8; and ARID1A) were also reported to be associated with the pathogenesis of FL [24]. Besides, gene mutations including *STAT6* and *RRAGC* were related to FL pathogenesis, which involving multiple pathways including mTOR and JAK/STAT pathways [25–27]. In recent studies, clinicogenetic risk models integrating genes for predicting prognosis of FL has been established [7, 8] and has showed the value as the prediction model in FL management. For germinal center-derived lymphoma, including FL, moving out of germinal center and local endothelial cells is essential for dissemination to other lymph nodes or tissues [28]. Previous studies showed that interaction of lymphoma cell with chemokine played an important role in the movement of lymphoma cell through endothelial cells [29–31] in chronic lymphocytic leukemia and classical Hodgkin lymphoma. Chemokines modulated chemotaxis in the migration and pathogenesis of FL cells was also presented in previous studies [32, 33]. In this study, among the three modules identified, the turquoise module showed significantly association with FL tumors. Functional analysis showed that genes in turquoise module was enriched in cell chemotaxis, extracellular matrix organization and extracellular structure organization. Also, the signaling pathway and downstream factors of the eight hub genes identified from the turquoise module were further explored by retrieving KEGG database and previous reports (Additional file 1). Results of our study further supported that pathway of cell interaction with chemokine, as well as other pathways including PI3K/AKT pathway and NF-kappa B signaling pathway, may be closely related to the development of FL.

In this study, we identified that *CCL19* was significantly associated with survival for FL patients treated with rituximab in combination with chemotherapy. *CCL19* encoded cytokine that involved in immunoregulatory and inflammatory processes. CCL19 specifically binds to chemokine receptor CCR7. *CCR7* and *CCL19* played an important role in organizing thymic architecture and function, lymph-node homing of naive and regulatory T cell, as well as homeostasis and inflammation-induced lymph-node-bound migration of dendritic cells, which indicated that *CCR7* and *CCL19* involved in the homeostasis, immune surveillance, and tumor formation [34]. In previous studies, *CCR7/CCL19* was reported to be associated with some types of cancer. CCR7 chemokine receptor binds to the ligand CCL19/CCL21 and promotes lymphogenesis and metastasis in breast cancer [35]. *CCL19* overexpression significantly inhibited gastric cancer cell proliferation and tumor growth through CCL19/CCR7/AIM2 pathway [36]. Also, *CCL19* overexpression is associated with malignant transformation in cervical cancer [37]. For lymphoma, O'Connor et al. reported that CCL19-CCR7 interactions may contributed to the increasing risk of age-related central nervous system lymphoma [38]. In T cell lymphoma, higher expression of *CCR7* was associated with distant metastasis as well as tumor cell migration in vitro and the underlying mechanism might be associated with PI3K/AKT signaling pathway [39].

In our study, *CCL19* was found to be overexpressed in different FL cell lines utilizing public database analysis. Previous studies have showed that strong in vitro chemotactic activities of CCL19 for T cells and DCs and might activate a LTα1β2-dependent pathway of normal and pathological lymphoid tissue formation [40]. In addition, CCL19 mRNA overexpression was related to higher survival rate. Also, elevated serum CCL19 concentration was associated with longer PFS and OS. The protective prognostic value of higher *CCL19* expression level had been validated at both the transcription and protein concentration in two independent FL patient cohorts. In the analysis of immune cell infiltration in tumor microenvironment, the expression of *CCL19* was associated with macrophages M1 and monocyte. The protective effect of *CCL19* overexpression might be explained from the mechanism of CCL19 and the formation of FL. CCL19 was produced by T-zone fibroblastic reticular cells and are essential for the formation and maintenance of the T-cell zone in lymphoid organs, as well as T cells and DCs peripheral recruitment. Therefore, elevated *CCL19* expression could induce the function of T-zone reticular cells and recruit T cells and DCs to tumor tissues [41]. Previous studies have reported the chemotaxis function of CCL19 for macrophages M1 [42]. Also, decreased subpopulations of CD4+ /CD8+ T cells, macrophages and dendritic cells in patients are associated with FL transformation and are predictors of worse survival [43, 44], which was consistent with the result of TIIC subtype analysis in our study.

In B cell lymphomas, including FL, DCs have showed to be correlated with better prognosis, suggesting that DCs may act against tumor cells in lymphoma [45–47]. Although in this study, the DC proportion has no significant correlation with the expression of *CCL19*, it is still worthwhile to exploring the function of *CCL19* and DC cells for potential clinical treatment. In fact, new generation of CAR-T cell has been engineered to expressed IL-7 and CCL19 to elevate anti-tumor efficacy [48]. Moreover, dendritic cell-based active immunotherapies [49] and macrophage checkpoint inhibitor Hu5F9-G4 have showed efficacy in FL [50]. Result of this study indicated that *CCL19* could act as a biomarker to identify FL patients who may have inferior efficacy derived from first-line immunotherapy. Considering the DC and macrophages recruitment capability of *CCL19*, it is worthwhile to explore whether these patients could benefit from dendritic cell-based active or macrophage-mediated immunotherapies for stimulating the innate immune system.

In this study, we sought to identify biomarkers for FL and further explore the prognostic role of *CCL19* in FL. There are some limitations in our study. The OS data was not mature enough for multivariate COX analysis in CHCAMS cohort. Longer follow-up time was warranted. The mechanisms of immune reactions induced by *CCL19* were not explored in our study and need to be further investigated to explore the underlying mechanism.

## Conclusion

*CCL19* might be a potential survival biomarker for FL treated with first-line chemoimmunotherapy. In vitro, in vivo, and clinical studies are needed to be explored the underlying interaction and regulation mechanism of *CCL19* for FL in our future studies.

## Supplementary Information

**Additional file 1.** Supplementary figures, tables and supporting information.

## Data Availability

Dataset GSE65135 and GSE119214 are publicly available, which can be found at https://www.ncbi.nlm.nih.gov/gds/. Data of CHCAMS cohort can be obtained from the corresponding authors under reasonable requirement.

## Abbreviations

FL: Follicular lymphoma
NHL: Non-Hodgkin lymphoma
PFS: Progression-free survival
OS: Overall survival
WGCNA: Weighted gene co-expression network analysis
RNA-seq: RNA sequencing
GEO: Gene Expression Omnibus
DEG: Differential expressed gene
R-CHOP: Rituximab plus cyclophosphamide, doxorubicin, vincristine, and prednisone
GO: Gene Ontology
KEGG: Kyoto Encyclopedia of Genes and Genomes
GSEA: Gene Set Enrichment Analysis
TOM: Topological overlap matrix
PPI: Protein–protein interaction
ELISA: Enzyme-linked immunosorbent assay
TIIC: Tumor-infiltrating immune cell
DC: Dendritic cell
