# Supplementary material for: Low CCL19 expression is associated with adverse clinical outcomes for follicular lymphoma patients treated with chemoimmunotherapy
Source: J Transl Med. 2021 Sep 20;19:399. doi: 10.1186/s12967-021-03078-9 (PMC8454033; doi:10.1186/s12967-021-03078-9)
Supplement: Supplementary file 1 — Additional file 1. Supplementary figures, tables and supporting information. [file 12967_2021_3078_MOESM1_ESM.docx]

**Supplementary Material**

**Low CCL19 Expression is associated with Adverse Clinical Outcomes for Patients with Advanced Stage Follicular Lymphoma**

Yu Zhou, Shasha Wang, Yunxia Tao, Haizhu Chen, Yan Qin, Xiaohui He, Shengyu Zhou, Jianliang Yang, Sheng Yang, Lin Gui, Ning Lou, Zhishang Zhang, Jiarui Yao, Xiaohong Han, Yuankai Shi

**Content**

**Supplementary Table S1. Patient characteristics of validation cohorts. 2**

**Supplementary Fig S1. Gene Set Enrichment Analysis of genes in dataset GSE65135.3**

**Supplementary Fig S2. Cluster dendrogram identifying outlined samples in WGCNA analysis of GSE65135.4**

**Supplementary Fig S3.** **Scale independence and mean connectivity under different soft threshold powers for scale-free network in WGCNA analysis of GSE65135. 5**

**Supplementary Fig S4. Network plot displaying pathways enriched in GO (A) and KEGG (B) analysis for genes of turquoise module in WGCNA analysis.****6**

**Supplementary** **Fig S5.** **(A) Top 50 genes ranked by weighted degree in turquoise module of GSE65135.** **(B) PPI network of the 8 hub genes.** **7**

**Supplementary** **Fig S6. mRNA expression of eight hub genes among different tumors in Oncomine database.** **8**

**Supplementary Fig S7. Cutoff value of eig****ht hub genes for GSE119214.** **9**

**Supplementary Fig S8. Gene Set Enrichment Analysis of genes grouped by *CCL19* expression in dataset GSE119214. 10**

**Supplementary Fig S9. Cutoff value of CCL19 for CHCAMS cohort.** **11**

**Supplementary Fig S10. Multivariate COX analysis of progression-free survival in CHCAMS cohort. 12**

**Supplementary Information. Signaling pathway and downstream factors of identified hub genes in GSE65135. 13**

**Supplementary Table S1. Patient characteristics of validation cohorts.**

| **Characteristics** | **GSE119214 (n=137)** | **CHCAMS cohort (n=32)** |
| --- | --- | --- |
| Sample source | Pre-treatment FFPE samples | Pre-treatment serum samples |
| Gender |  |  |
| Male | NA | 16 (50.0%) |
| Female | NA | 16 (50.0%) |
| Age, median (range) | NA | 47 (26-72) |
| Age over 60 |  |  |
| Yes | NA | 8 (25.0%) |
| No | NA | 24 (75.0%) |
| Rituximab maintenance |  |  |
| Yes | NA | 10 (31.2%) |
| No | NA | 22 (68.8%) |
| FLIPI group |  |  |
| Low-risk | NA | 9 (28.1%) |
| Intermediate-risk | NA | 15 (46.9%) |
| High-risk | NA | 8 (25.0%) |
| PFS, median (range) (months) | 83.8 (0.6-170.4) | 46.5 (6.0-76.0) |
| OS, median (range) (months) | 102 (1.3-170.4) | 51.0 (12.0-98.0) |
| Median follow-up time (months) | NA | 58.0 |

**Abbreviations:** FFPE, formalin-fixed and paraffin-embedded; FLIPI, Follicular Lymphoma International Prognostic Index; PFS, progression-free survival; OS, overall survival.

**Supplementary Fig S1. Gene Set Enrichment Analysis** **of genes in dataset GSE65135.**

**
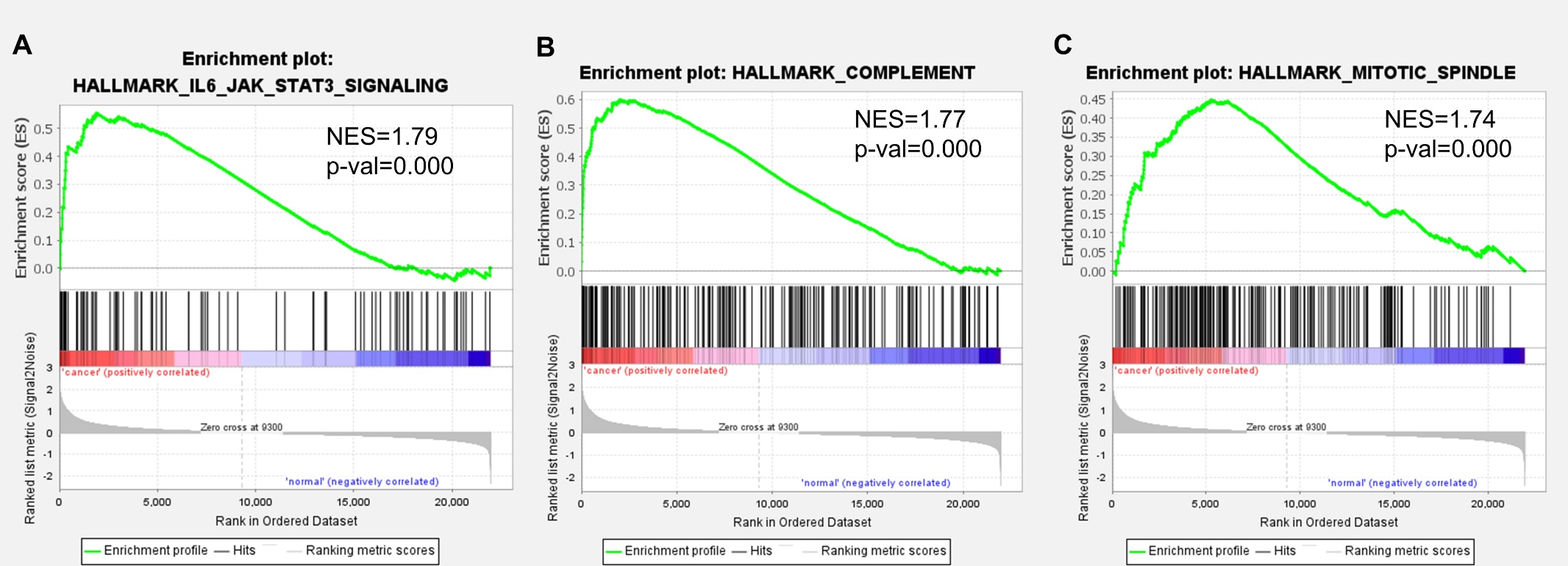
**

**Supplementary Fig S2. Cluster dendrogram identifying outlined samples in WGCNA analysis** **of** **GSE65135.**


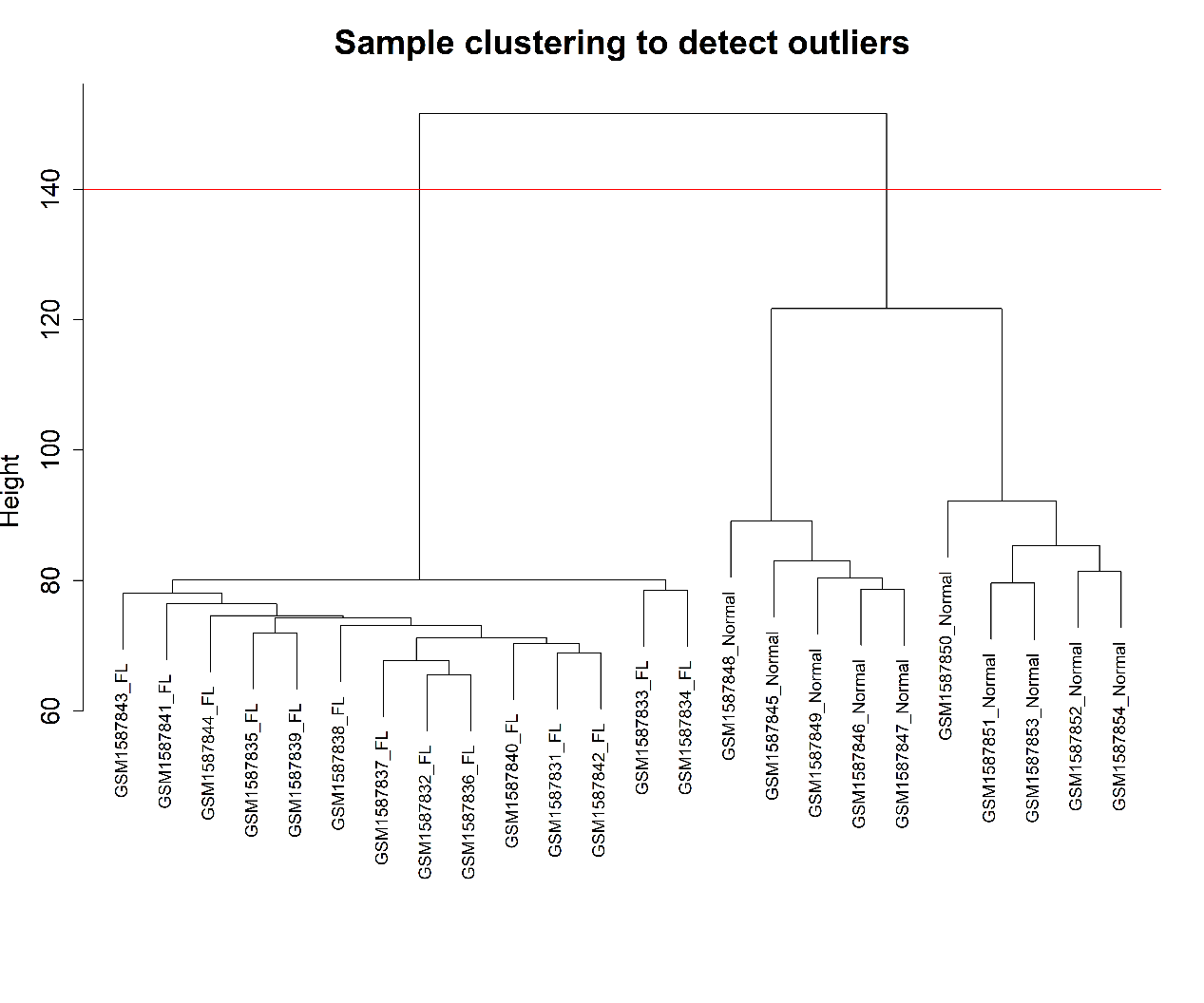


**Supplementary Fig S3.** **Scale independence and mean connectivity under different soft threshold powers for scale-free network in WGCNA analysis of GSE65135.** Soft thresholding power was set at 6 to ensure a scale-free network, with scale-free R^2^ >0.8 (in the left graph) and mean k >100 (in the right graph).


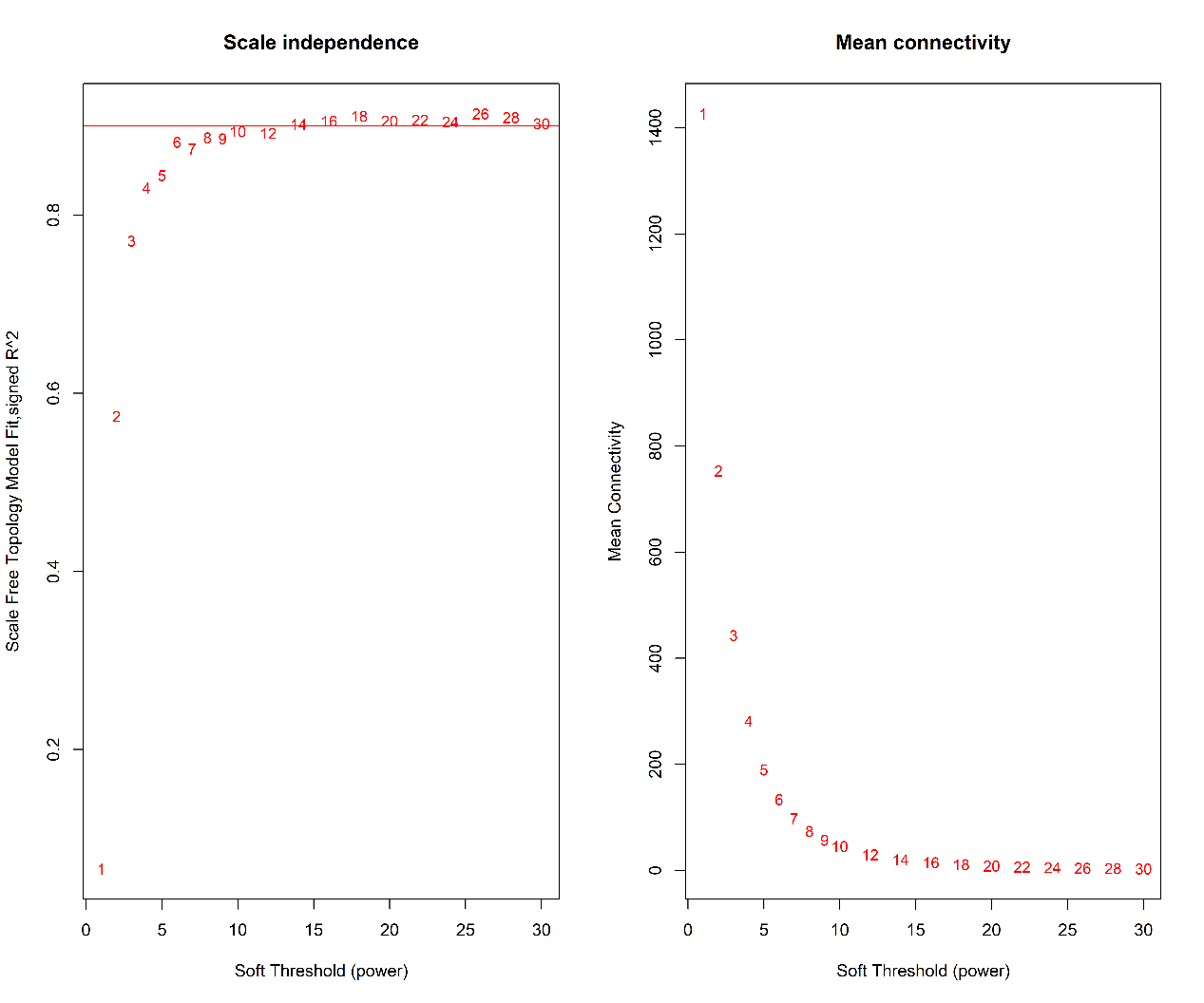


**Supplementary Fig S4. Network plot displaying pathways enriched in GO (A) and KEGG (B) analysis** **for genes of** **turquoise module in WGCNA analysis.**


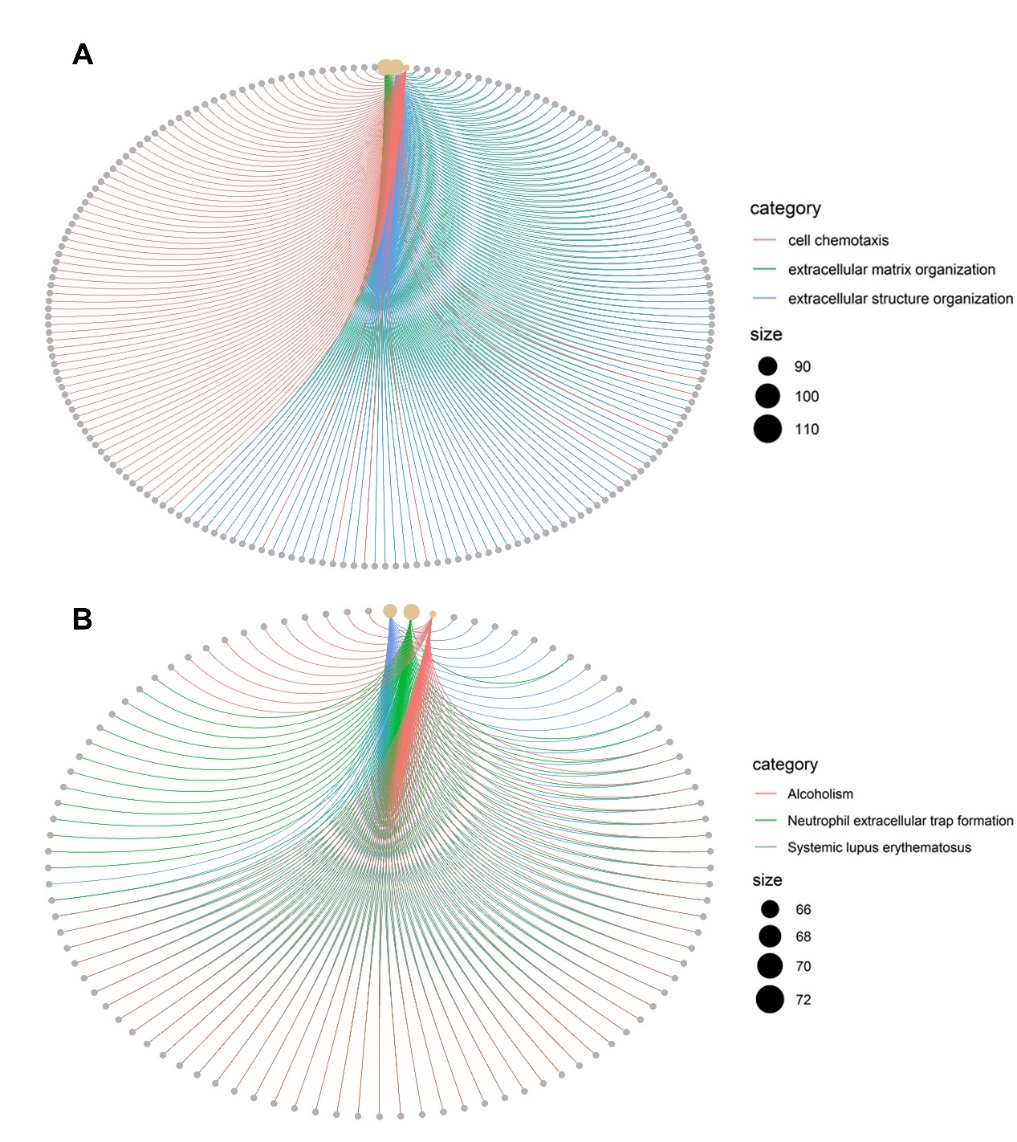


**Supplementary** **Fig S5.** **(A) Top 50 genes ranked by weighted degree in turquoise module** **of GSE65135.** Color of nodes are ordered according to their degree with red nodes representing higher degree while yellow nodes representing lower degree. **(B) PPI network of the 8 hub genes.**


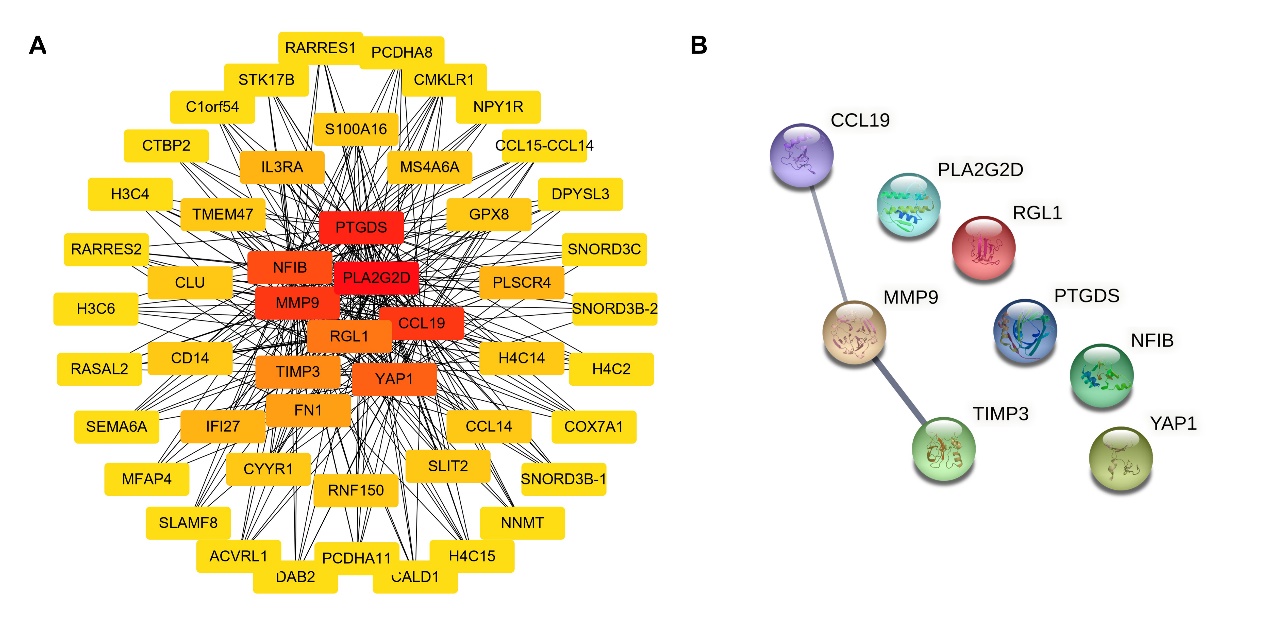


**Supplementary** **Fig S6. mRNA expression of hub genes among different tumors in Oncomine database.** Graph showed the datasets which mRNA of the hub genes were significantly overexpressed (red) and down-regulated (blue). The threshold for p value were 0.01. Numbers in each cell indicated the number of analyses that met the threshold within each cancer type.

Note: *Cell color is determined by the best gene rank percentile for the analyses within the cell.


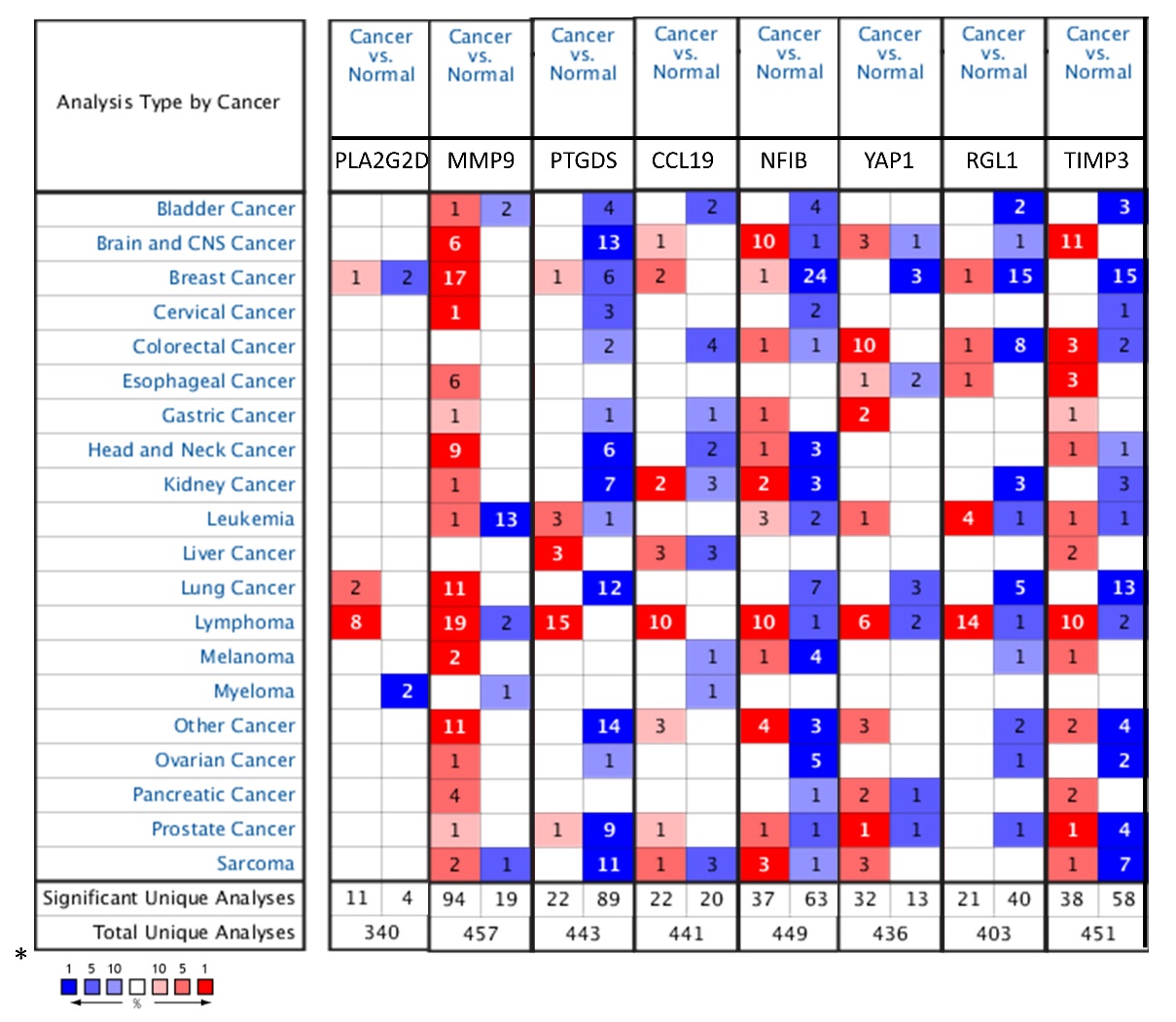


**Supplementary Fig S7. Cutoff value of eight hub genes for GSE119214.**  Samples were grouped into high expression and low expression groups based on the cutpoint of gene expression value, with expression value lower than cutpoint defined as low expression group and expression value higher than cutpoint defined as high expression group.


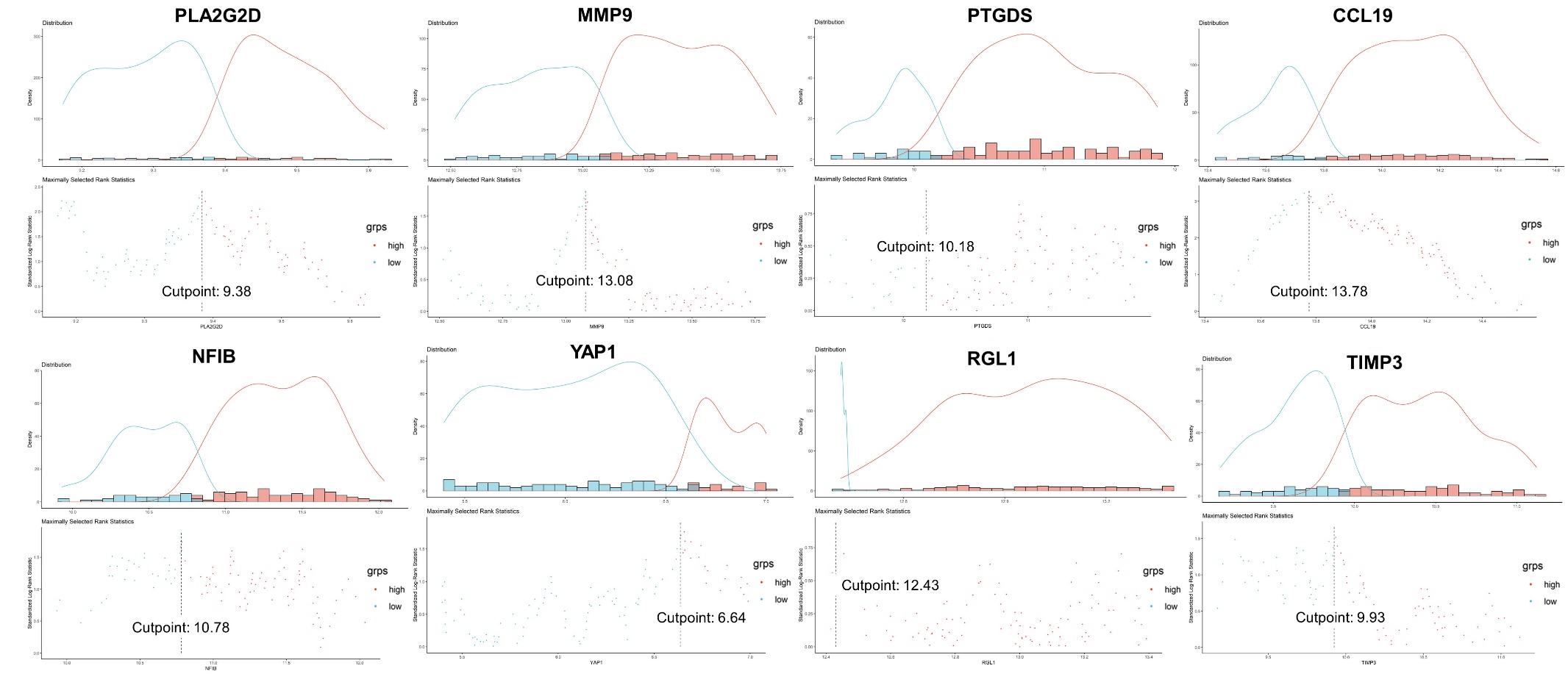


**Supplementary Fig S8.** **Gene Set Enrichment Analysis of genes** **grouped by *CCL19* expression in dataset** **GSE119214. Top 3 enriched pathways in *CCL19* low expression group(A-C); Top 3 enriched pathways in *CCL19* high expression group (D-F).**

**
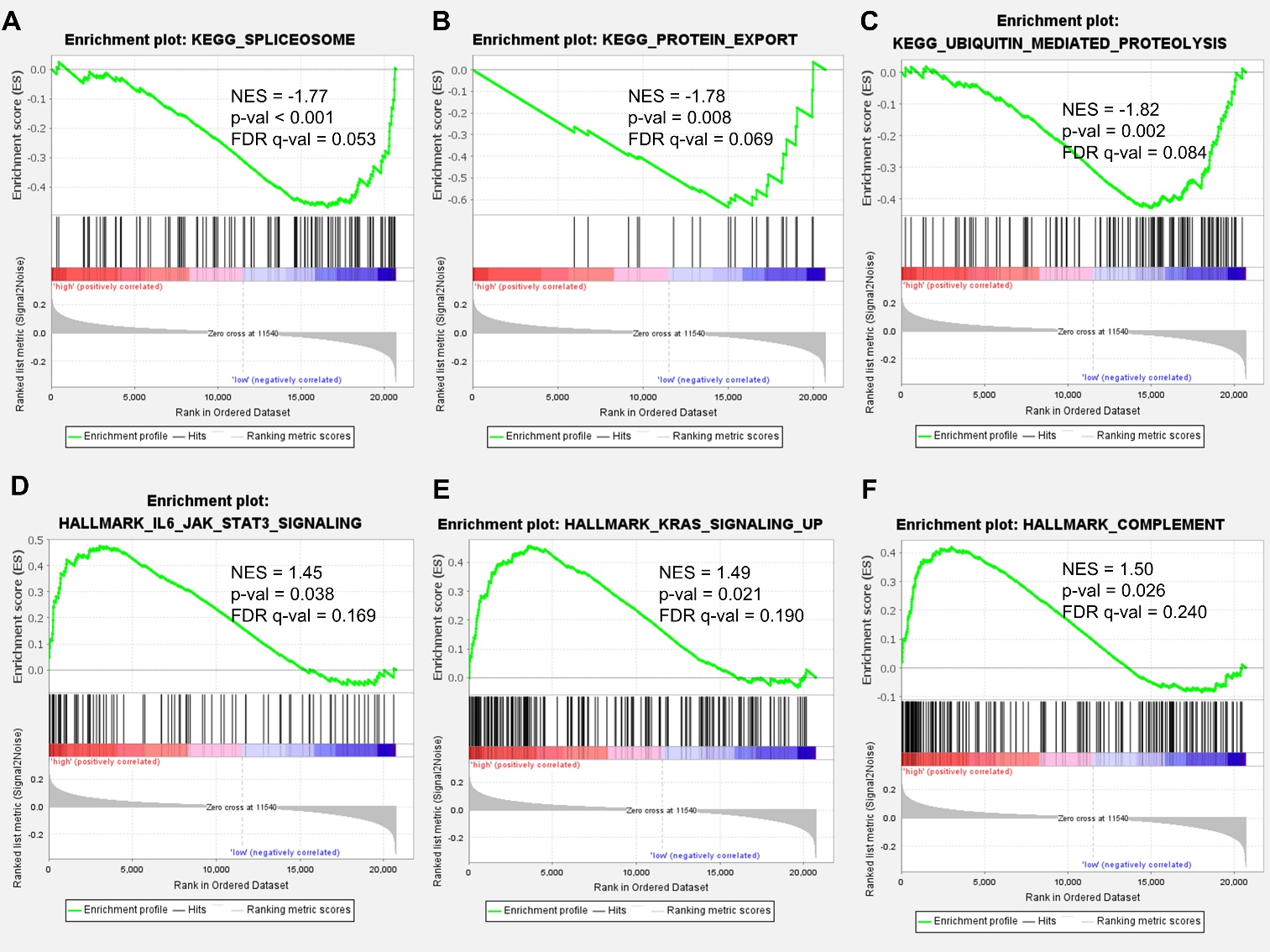
**

**Supplementary Fig S9. Cutoff value of CCL19 for CHCAMS cohort.** Samples were grouped into high expression and low expression groups based on the cutpoint of CCL19 concentration, with expression value lower than 0.94 defined as low expression group and expression value hihger than 0.94 defined as high expression group.


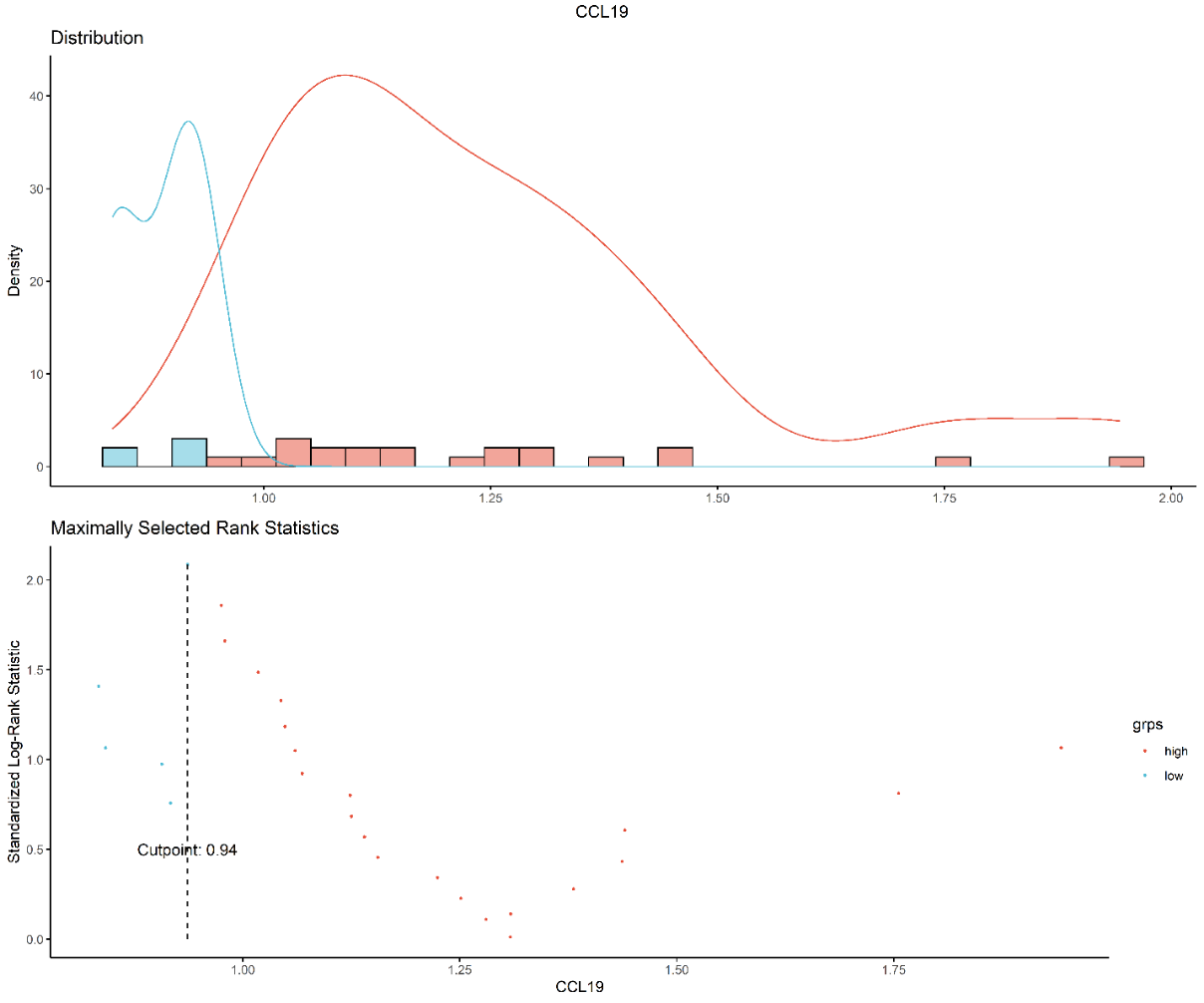


**Supplementary Fig S10. Multivariate COX analysis of progression-free survival in CHCAMS cohort.**

**Abbreviations:** FLIPI, Follicular Lymphoma International Prognostic Index; HR, hazard ratio; CI, confidence interval.

**
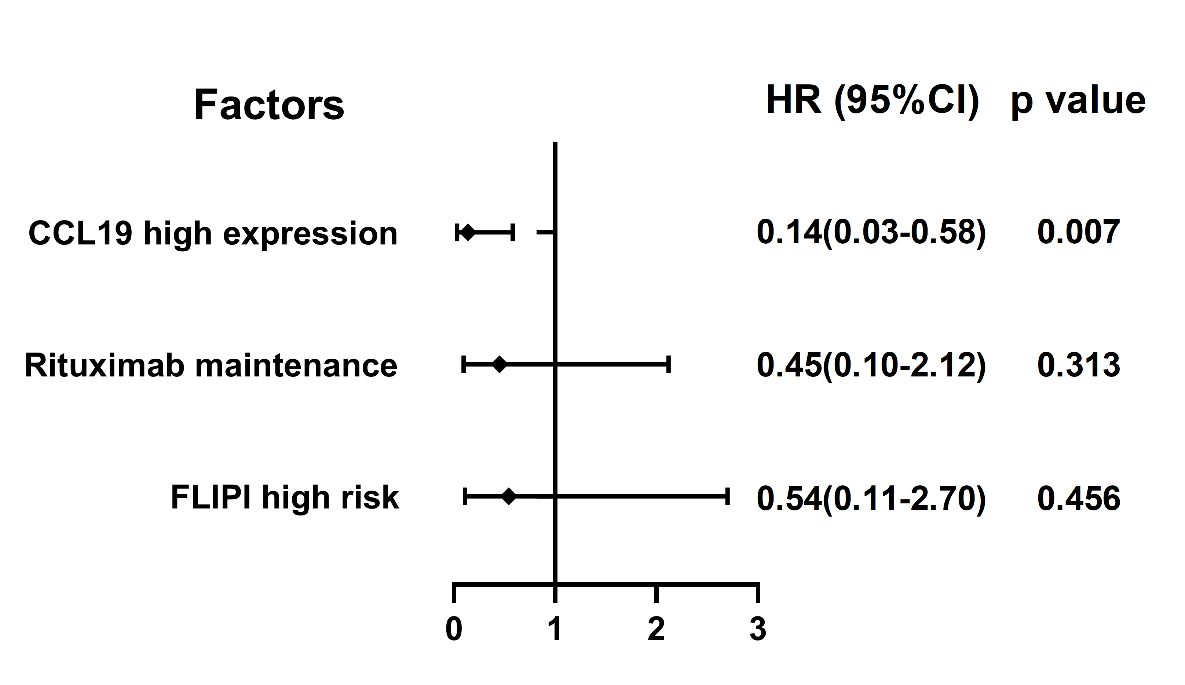
**

**Supplementary information**

**Signaling pathway and downstream factors of identified hub genes** **in GSE65135**

**1.** **CCL19 (C-C motif chemokine ligand 19)**

1.1 Viral protein interaction with cytokine and cytokine receptor pathway

Previous studies showed that chemokine receptors U51 (encoded by herpesvirus 6 [HHV-6] and HHV-7) and U12 (encoded by HHV-7). CCL19 acts as ligand for U51 and U12, and thus regulates the viral protein interaction with cytokine and cytokine receptor pathway [1, 2] and affect different aspects of immunity.


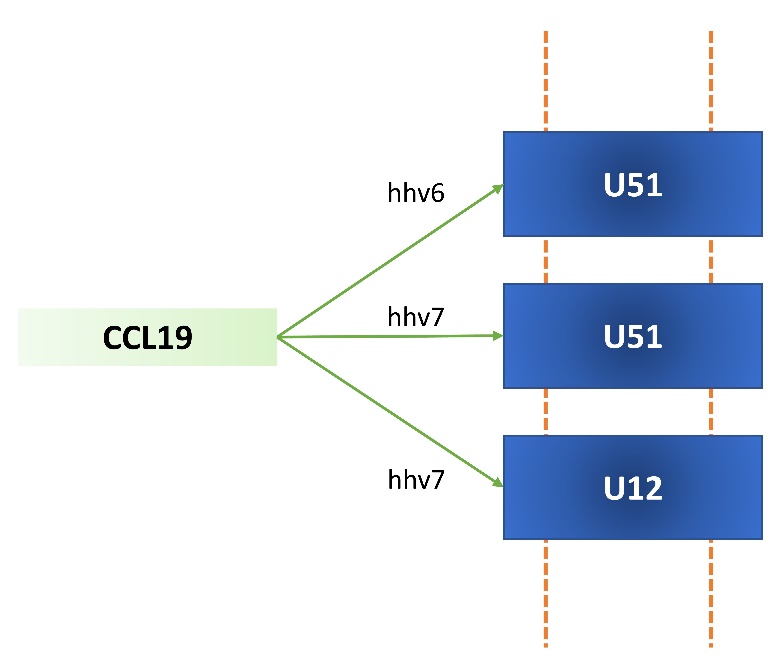


1.2 Cytokine-cytokine (CC) receptor interaction pathway and chemokine signaling pathway

CCL19 belongs to CC subfamily. As soluble extracellular protein, CCL19 can bind to receptors including CCR11 and CCR7 on the cell surface of target cells and thus induces the downstream innate as well as adaptive inflammatory host defenses, cell growth, differentiation, cell death, angiogenesis, and development and repair processes aimed at the restoration of homeostasis[3, 4].


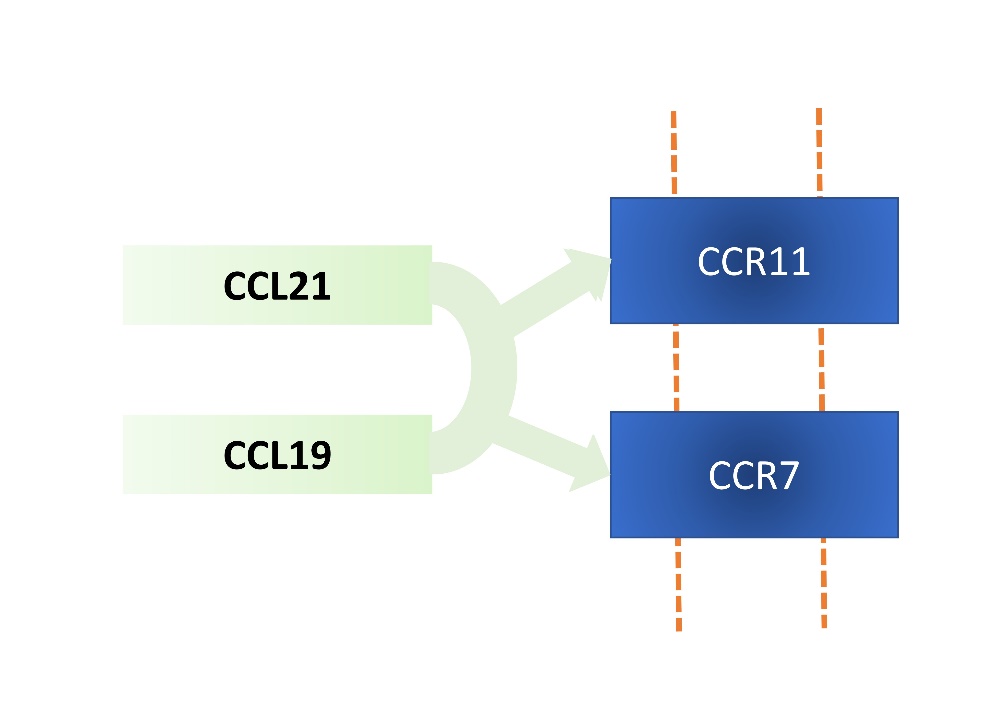


1.3 NF-kappa B signaling pathway

CCL19 is the down-stream molecule of NF-kappa B pathway, mainly involving in lymphoid-tissue homing[5].

1.4 Other reported pathways and downstream factors

(1) Phosphoinositide-3 kinase (PI3K)/AKT pathway: Previous study indicated that CCL19 and its receptor CCR7 functioned as an upstream factor of the AKT pathway contributing to the expression of GATA2, promoting trophoblast migration, and invasion via MMP2[6]. CCL9-CCR7 activates PI3K to promote invasion and survival of SCCHN cells, and Cdc42 might be the downstream factor involved and regulated by CCL19-CCR7[7].

(2) RhoA/ Rho-associated kinase (ROCK) pathway: Previous study reported that RhoA, proline-rich tyrosine kinase-2 (Pyk2) and cofilin was activated by CCL19 and increased RhoA, Pyk2 and cofilin activity was eliminated by CCR7mAb, indicating that CCL19-CCR7 acted as upstream regulating factor[8].

**2. TIMP3 (TIMP metallopeptidase inhibitor 3)**

2.1 MicroRNAs in cancer

TIMP3 is one of the important tumor suppressor gene. In lung cancer, TIMP3 is the target of miR-221/222 and regulates the downstream PI3K/AKT pathway to mediate the tumorigenesis and tumor proliferation[9].

2.2 Proteoglycans in cancer

TIMP3 binds to matrix metalloproteinases 2 (MMP2) and MMP9, and further regulates the downstream factor including VEGFA and VEGFR2, therefore involving in the process of tumor angiogenesis[10].

**3.MMP9 (matrix metallopeptidase 9)**

MMP9 mainly involves the pathway of proteoglycans in cancer. MMP9 acts as the downstream factor of TIMP3 and THBS1, and further regulates the downstream factor including VEGFA and VEGFR2, therefore involving in the process of tumor angiogenesis[10].

**4. PLA2G2D (phospholipase A2 group IID)**

PLA2G2D secreted member of the phospholipase A2 family, and mainly involved in multiple metabolism pathways including glycerophospholipid metabolism, Ether lipid metabolism, arachidonic acid metabolism, etc. Study indicated that the upregulated expression of PLA2G2D was a potential biomarker of adaptive resistance to immune checkpoint blockade[11] .

**5.** **NFIB (nuclear factor I B)**

NFIB encodes a prominent transcription factor. NFIB mainly involved in cell migration and potentially invasion in different types of cancer. Study showed that NFIB drove the chromatin changes in human SCLC cell lines, and further promoted the metastasis procedure[12]. MiR-302a was found to be its upstream regulator in colorectal cancer[13] and miR-205-5p regulates NFIB in breast cancer[14].

**6. YAP1 (Yes1 associated transcriptional regulator)**

YAP is one of the important factors of Hippo signaling pathway, which is an evolutionarily conserved signaling pathway. The dysfunction of Hippo pathway leads to the increased activity of YAP/TAZ with an underphosphorylated form in the nucleus. YAP/TAZ further interacts with transcription factors including transcription enhancers activation domain (TEAD) family members, which can drive cancer cell survival-related gene expression and tumor growth.[15-17]

In fact, YAP1/TAZ/TEAD has been treated as an important pro-cancer signaling pathway and found to be related with multi-types of cancer including small cell lung cancer [18], non-small cell lung cancer [19]and esophageal squamous cell carcinoma [20]. Preclinical trials targeting YAP1/TAZ/TEAD are ongoing[15].

**7. RGL1 (ral guanine nucleotide dissociation stimulator like 1)**

RGL1 is one of the highly related RalGEFs and serves as RAS effector in Ras signaling pathway. RGL1 further regulates the Ral activation and has an impact on the downstream signaling pathways including JNK and NF-κB and further activate Ras-driven cancer invasion[21, 22].

**8.** **PTGDS (prostaglandin D2 synthase)**

PTGDS is one of the prostaglandins (PGs) family involved in arachidonic acid metabolism, which derived from arachidonic acid through the cyclooxygenase (COX) pathway. PTGDS involves in the metabolic process of PGH2 to PGJ2 and synthesizes PGD2, which is also shown to contribute to SOX9 nucleus translocation and further inhibits the migration and invasion of cancer cells.[23] Lack of PTGDS leads to changes in the function of tumor endothelial cells and further reduce the tumor cell apoptosis[24]. Interestingly, in previous study, YAP showed to inhibit the expression of lipocalin-type PTGDS (L-PTGDS) and thus activated the proliferation of gastric cancer stem cell[25] .

***References:***

1. Tadagaki K, Yamanishi K, Mori Y. Reciprocal roles of cellular chemokine receptors and human herpesvirus 7-encoded chemokine receptors, U12 and U51. *J Gen Virol.* 2007; **88**:1423-1428. doi: 10.1099/vir.0.82665-0

2. Fitzsimons CP, Gompels UA, Verzijl D, Vischer HF, Mattick C, Leurs R, Smit MJ. Chemokine-directed trafficking of receptor stimulus to different g proteins: selective inducible and constitutive signaling by human herpesvirus 6-encoded chemokine receptor U51. *Mol Pharmacol.* 2006; **69**:888-898. doi: 10.1124/mol.105.015222

3. Khoja H, Wang G, Ng CT, Tucker J, Brown T, Shyamala V. Cloning of CCRL1, an orphan seven transmembrane receptor related to chemokine receptors, expressed abundantly in the heart. *Gene.* 2000; **246**:229-238. doi: 10.1016/s0378-1119(00)00076-7

4. Birkenbach M, Josefsen K, Yalamanchili R, Lenoir G, Kieff E. Epstein-Barr virus-induced genes: first lymphocyte-specific G protein-coupled peptide receptors. *J Virol.* 1993; **67**:2209-2220. doi: 10.1128/jvi.67.4.2209-2220.1993

5. Rossi DL, Vicari AP, Franz-Bacon K, McClanahan TK, Zlotnik A. Identification through bioinformatics of two new macrophage proinflammatory human chemokines: MIP-3alpha and MIP-3beta. *J Immunol.* 1997; **158**:1033-1036. doi:

6. Luan X, Li S, Zhao J, Zhai J, Liu X, Chen ZJ, Li W, Du Y. Down-regulation of CCR7 via AKT pathway and GATA2 inactivation suppressed trophoblast migration and invasion in recurrent spontaneous abortion†. *Biol Reprod.* 2020; **102**:424-433. doi: 10.1093/biolre/ioz172

7. Zhao ZJ, Liu FY, Li P, Ding X, Zong ZH, Sun CF. CCL19-induced chemokine receptor 7 activates the phosphoinositide-3 kinase-mediated invasive pathway through Cdc42 in metastatic squamous cell carcinoma of the head and neck. *Oncol Rep.* 2011; **25**:729-737. doi: 10.3892/or.2010.1109

8. Xu Z, Zheng X, Yang L, Liu F, Zhang E, Duan W, Bai S, Safdar J, Li Z, Sun C. Chemokine receptor 7 promotes tumor migration and invasiveness via the RhoA/ROCK pathway in metastatic squamous cell carcinoma of the head and neck. *Oncol Rep.* 2015; **33**:849-855. doi: 10.3892/or.2014.3631

9. Garofalo M, Quintavalle C, Romano G, Croce CM, Condorelli G. miR221/222 in cancer: their role in tumor progression and response to therapy. *Curr Mol Med.* 2012; **12**:27-33. doi: 10.2174/156652412798376170

10. Qi JH, Ebrahem Q, Moore N, Murphy G, Claesson-Welsh L, Bond M, Baker A, Anand-Apte B. A novel function for tissue inhibitor of metalloproteinases-3 (TIMP3): inhibition of angiogenesis by blockage of VEGF binding to VEGF receptor-2. *Nat Med.* 2003; **9**:407-415. doi: 10.1038/nm846

11. Cindy Yang SY, Lien SC, Wang BX, Clouthier DL, Hanna Y, Cirlan I, Zhu K, Bruce JP, El Ghamrasni S, Iafolla MAJ, et al. Pan-cancer analysis of longitudinal metastatic tumors reveals genomic alterations and immune landscape dynamics associated with pembrolizumab sensitivity. *Nat Commun.* 2021; **12**:5137. doi: 10.1038/s41467-021-25432-7

12. Denny SK, Yang D, Chuang CH, Brady JJ, Lim JS, Grüner BM, Chiou SH, Schep AN, Baral J, Hamard C, et al. Nfib Promotes Metastasis through a Widespread Increase in Chromatin Accessibility. *Cell.* 2016; **166**:328-342. doi: 10.1016/j.cell.2016.05.052

13. Sun L, Fang Y, Wang X, Han Y, Du F, Li C, Hu H, Liu H, Liu Q, Wang J, et al. miR-302a Inhibits Metastasis and Cetuximab Resistance in Colorectal Cancer by Targeting NFIB and CD44. *Theranostics.* 2019; **9**:8409-8425. doi: 10.7150/thno.36605

14. Chen H, Yu C, Shen L, Wu Y, Wu D, Wang Z, Song G, Chen L, Hong Y. NFIB functions as an oncogene in estrogen receptor-positive breast cancer and is regulated by miR-205-5p. *Pathol Res Pract.* 2020; **216**:153236. doi: 10.1016/j.prp.2020.153236

15. Felley-Bosco E, Stahel R. Hippo/YAP pathway for targeted therapy. *Transl Lung Cancer Res.* 2014; **3**:75-83. doi: 10.3978/j.issn.2218-6751.2014.02.03

16. Kandasamy S, Adhikary G, Rorke EA, Friedberg JS, Mickle MB, Alexander HR, Eckert RL. The YAP1 Signaling Inhibitors, Verteporfin and CA3, Suppress the Mesothelioma Cancer Stem Cell Phenotype. *Mol Cancer Res.* 2020; **18**:343-351. doi: 10.1158/1541-7786.Mcr-19-0914

17. Zhao B, Ye X, Yu J, Li L, Li W, Li S, Yu J, Lin JD, Wang CY, Chinnaiyan AM, et al. TEAD mediates YAP-dependent gene induction and growth control. *Genes Dev.* 2008; **22**:1962-1971. doi: 10.1101/gad.1664408

18. Rudin CM, Poirier JT, Byers LA, Dive C, Dowlati A, George J, Heymach JV, Johnson JE, Lehman JM, MacPherson D, et al. Molecular subtypes of small cell lung cancer: a synthesis of human and mouse model data. *Nat Rev Cancer.* 2019; **19**:289-297. doi: 10.1038/s41568-019-0133-9

19. Wang Y, Dong Q, Zhang Q, Li Z, Wang E, Qiu X. Overexpression of yes-associated protein contributes to progression and poor prognosis of non-small-cell lung cancer. *Cancer Sci.* 2010; **101**:1279-1285. doi: 10.1111/j.1349-7006.2010.01511.x

20. Muramatsu T, Imoto I, Matsui T, Kozaki K, Haruki S, Sudol M, Shimada Y, Tsuda H, Kawano T, Inazawa J. YAP is a candidate oncogene for esophageal squamous cell carcinoma. *Carcinogenesis.* 2011; **32**:389-398. doi: 10.1093/carcin/bgq254

21. Zago G, Veith I, Singh MK, Fuhrmann L, De Beco S, Remorino A, Takaoka S, Palmeri M, Berger F, Brandon N, et al. RalB directly triggers invasion downstream Ras by mobilizing the Wave complex. *Elife.* 2018; **7**. doi: 10.7554/eLife.40474

22. Vigil D, Martin TD, Williams F, Yeh JJ, Campbell SL, Der CJ. Aberrant overexpression of the Rgl2 Ral small GTPase-specific guanine nucleotide exchange factor promotes pancreatic cancer growth through Ral-dependent and Ral-independent mechanisms. *J Biol Chem.* 2010; **285**:34729-34740. doi: 10.1074/jbc.M110.116756

23. Shyu RY, Wu CC, Wang CH, Tsai TC, Wang LK, Chen ML, Jiang SY, Tsai FM. H-rev107 regulates prostaglandin D2 synthase-mediated suppression of cellular invasion in testicular cancer cells. *J Biomed Sci.* 2013; **20**:30. doi: 10.1186/1423-0127-20-30

24. Ragolia L, Palaia T, Hall CE, Klein J, Büyük A. Diminished lipocalin-type prostaglandin D(2) synthase expression in human lung tumors. *Lung Cancer.* 2010; **70**:103-109. doi: 10.1016/j.lungcan.2010.01.011

25. Bie Q, Li X, Liu S, Yang X, Qian Z, Zhao R, Zhang X, Zhang B. YAP promotes self-renewal of gastric cancer cells by inhibiting expression of L-PTGDS and PTGDR2. *Int J Clin Oncol.* 2020; **25**:2055-2065. doi: 10.1007/s10147-020-01771-1
